# Supplementary material for: Analysis of PPARγ Signaling Activity in Psoriasis
Source: Int J Mol Sci. 2021 Aug 10;22(16):8603. doi: 10.3390/ijms22168603 (PMC8395241; doi:10.3390/ijms22168603)
Supplement: Supplementary file 1 [file ijms-22-08603-s001.zip › Supplemental materials_Analysis of PPARg signaling activity in psoriasis/Pathway models/Models images and html files/PPARG inhibits phenotypes assotiated with psoriasis/142683.html]

scar formation


# Cell Process scar formation

|  |  |
| --- | --- |
| URN | urn:agi-cellproc:Cicatrization |
| Total Entities | 0 |
| Connectivity | 2462 |
| Name | scar formation |

---

|  |  |
| --- | --- |
| Pathway | PPARG inhibits phenotypes assotiated with psoriasis |
|  | efects selected |

---

|  |  |
| --- | --- |
| MedScan ID | 4000519 |

---

|  |  |
| --- | --- |
| Alias | appearances of the scar |
|  | Scar formations |
|  | Scarrings |
|  | appearance of the scars |
|  | cicatrization |
|  | cicatrizations |
|  | appearance of the scar |
|  | Scar appearance |
|  | Scarring |
|  | scarifications |
|  | Scar formation |
|  | scarification |
|  | formation of scar |

---

|  |  |
| --- | --- |
| MeSH ID | M0514460 |

---
